# Associations of Interleukin-1*β* with *H. pylori*-Induced Gastric Atrophy and Syndrome of Dampness-Heat in the Spleen and Stomach in Subjects with *H. pylori*-Related Gastric Diseases

**DOI:** 10.1155/2020/6409485

**Published:** 2020-02-12

**Authors:** Yunzhan Zhang, Xu Chen, Lin Gong, Danyan Li, Yun-kai Dai, Shaoyang Lan, Qi Luo, Bin Chen, Weijing Chen, Ruliu Li, Ling Hu

**Affiliations:** ^1^Institute of Gastroenterology, Guangzhou University of Chinese Medicine, Guangzhou 510000, China; ^2^First Affiliated Hospital of Guangzhou University of Chinese Medicine, Guangzhou 510000, China

## Abstract

*H. pylori*-related gastric diseases (HPGD) are a series of gastric mucosal benign and malignant lesions associated with *H. pylori* infection. Exploring the pathogenesis of HPGD will be of great significance to prevent and treat gastric malignancy. Traditional Chinese medicine (TCM) syndrome is the essence of TCM, reflecting the state of whole body. Potential similarities of TCM syndrome may provide a new perspective in understanding development and treatment of diseases. To seek an early warning signal for gastric malignant pathology and similarities of TCM syndrome from the viewpoint of molecular biology, we examined the relationships among *H. pylori*, gastric pathology, and TCM syndrome and effects of Interleukin-1*β* (IL-1*β*) gene polymorphisms and expression on gastric pathology and TCM syndrome in HPGD. The results indicated that detection of *H. pylori* with differentiation of TCM syndrome may have a predictive function to gastric pathology. *H. pylori* may lead to gastric atrophy via enhancing IL-1*β* mRNA expression, and IL-1*β* mRNA overexpression in gastric mucosa may be one of the generality characteristics for *H. pylori*-negative subjects with syndrome of dampness-heat in the spleen and stomach.

## 1. Introduction

Cancer is a common public health problem in the world. World Health Organization (WHO) reported that more than 9.6 million people have died of cancer in 2018, and gastric cancer (GC) was in the third place of the death rate of cancer. The development of GC is a complex, multistep, and multifactorial process. In 1992, based on evidence from pathology and epidemiology studies, Correa [1] put forward a human model of gastric carcinogenesis, which was chronic gastritis; atrophy; intestinal metaplasia (IM); and dysplasia. Infection with *Helicobacter pylori* (*H. pylori*) played a crucial role in the initial stages of gastritis and atrophy. *H. pylori* are a type of Gram-negative and microaerophilic bacteria which can attach to the surface of gastric mucosa and stimulate epithelial cells directly or indirectly to induce inflammation. Thus, *H. pylori* are believed to be the main cause of chronic gastric diseases. These diseases are known as *H. pylori*-related gastric diseases (HPGD), which contain benign and malignant gastric disorders. However, not all subjects infected with *H. pylori* suffer from GC, implicating that there are other key factors in the process of GC.

Recently, genetic factors involved in DNA methylation and gene mutation are believed to contribute to GC development. Single nucleotide polymorphism (SNP) is one kind of mutations which might lead to abnormal gene expression and determine an individual's susceptibility to diseases. Inflammatory pathways run through all stages of HPGD. Interleukin 1*β* (IL-1*β*) is a proinflammatory mediator, which activates various immune and inflammatory responses in gastric mucosa after *H. pylori* infection [2]. *IL-1B*-511 (rs 16944) has been associated with various inflammatory and infectious disorders, including *H. pylori* infection [3], chronic atrophic gastritis [4–6], gastric ulcer [7], and gastric carcinoma [8]. However, to the authors' knowledge, there are scarce data regarding the relation of *IL-1B*-511 polymorphism and gastric IM and dysplasia. *IL-1B*-511 TT genotype has been reported to upregulate expression of IL-1*β* in gastric mucosa in healthy subjects [9]. Therefore, it is necessary to explore systematically the relationship between *IL-1B*-511/IL-1*β* mRNA and pathological changes of gastric mucosa from normal to carcinoma.

A one-sided conclusion is easily obtained when disease development is analysed only from a single factor (e.g., pathogenic, genetic, or environmental factors). Based on holism, traditional Chinese medicine (TCM) puts forward the concept of “syndrome” defined as the reaction to or interaction with environmental and pathogenic factors for the host. Clinical physicians differentiate patients' syndrome through comprehensive analysis of signs and symptoms, determining the cause, nature, and location of the illness and the patients' physical condition, as well as treatment [10]. To some extent, TCM syndrome seems to have higher meaning than disease for the body. It has been reported that there is a close relationship between TCM syndrome and pathological changes in gastric diseases [11–13]. Gastric histopathology is regarded as the essence of gastric diseases in micro, while TCM syndrome as the whole in macro. However, there are few researches on association of TCM syndrome and gene polymorphism.

In this study, we analysed the relationship between gastric histopathology and TCM syndrome, as well as the association of gastric histopathology/TCM syndrome with some host factors (age, gender, and *IL-1B*-511 genotypes) and *H. pylori*, aiming to provide an early warning signal for gastric malignant pathology and to seek similarities of TCM syndrome. In addition, IL-1*β* mRNA levels in gastric mucosa were examined to investigate the molecular mechanism of gastric pathological changes and TCM syndrome.

## 2. Materials and Methods

### 2.1. Study Population

All of the subjects were recruited consecutively from the Endoscopy centre of First Affiliated Hospital of Guangzhou University of Chinese medicine (Guangdong, China) from September 2011 to December 2016. They comprised subjects undergoing a routine health check-up that included gastroscopy and patients with gastric disabling symptoms diagnosed as gastric inflammatory, gastric ulcer, or GC by gastroscopy. General information and clinical manifestations of subjects were collected. Several biopsy specimens were obtained from the antrum or the lesion location in the stomach. These specimens were used for *H. pylori* detection, gastric mucosal histopathology, IL-1*β* gene polymorphisms, and mRNA detection. All samples were delinked and unidentified from their donors. Written informed consent was obtained from each individual before the study. All procedures followed in the study were approved by the Ethics Committee of the First Affiliated Hospital of Guangzhou University of Traditional Chinese Medicine.

### 2.2. Differentiation of TCM Syndrome

TCM syndrome of subjects was differentiated by two expert TCM doctors according to guiding principles of clinical research on Chinese traditional medicine and new medicine (trial edition) in 2002 [14], syndrome of dampness-heat in the spleen and stomach diagnose standards [15], spleen-qi deficiency syndrome diagnose standards [16], and WHO international standard terminologies on traditional medicine in the Western Pacific region [10]. Syndrome was identified as follows: (1) Syndrome of dampness-heat in the spleen and stomach (DHSS): main symptoms include yellow dense and slimy fur and sticky stool; secondary symptoms include stomach fullness, nausea, bitter and sticky taste, heaviness sensation of the body, thirst with a little intake of fluid, and poor appetite. (2) Liver-stomach disharmony syndrome (LSD): main symptoms include epigastric and hypochondriac dispending pain, stuffiness sensation in the chest with frequent sighing, and symptoms linked to mood; secondary symptoms include belching, gastric upset, acid vomiting, irritability, white slippery or thin yellow fur, and string-like pulse. (3) Spleen-qi deficiency syndrome (SQD): main symptoms include anorexia with indigestion, fatigue, postprandial abdominal distension, and pale tongue with fat or teeth-marked tongue; secondary symptoms include lassitude, bland taste in mouth with no thirst, sallow complexion, loose stool, and weak and thin pulse. (4) Syndrome of blood stasis in the stomach collateral (BSSC): main symptoms include epigastric stabbing pain that is aggravated by pressure in the epigastric region, dark red tongue, and purple spots on the tongue; secondary symptoms include greyish complexion and string-like and rough pulse. TCM syndrome is confirmed for each subject in the condition of two main symptoms or one main symptom with two secondary symptoms. If two or more than two kinds of syndromes occur in one subject, the main syndrome will be judged by subsequent discussion. In addition, we set nonsyndrome (NON): the subjects have no uncomfortable symptoms or abnormal signs, with pale red tongue, thin white fur, and no obvious abnormal pulse.

### 2.3. *H. pylori* Detection and Assessment of Gastric Histopathology

Different methods for detection of *H. pylori* infection have different sensitivities and specificities [17, 18]. To improve the accuracy for the diagnosis of *H. pylori* infection, rapid urease test (Guangzhou Beisiqi Reagent Co., LTD, Guangdong, China) and methylene blue staining (Guangzhou Chemical Reagent Factory, Guangdong, China) were performed in this study. The subject would be classified as *H. pylori* infection if at least one of the two methods was positive. Histopathological evaluation was performed by the hematoxylin and eosin method and examined by a single pathologist blinded to the participants' other information. According to the update Sydney system [19] and Morson's criteria of grading gastric dysplasia [20], degrees of gastric inflammation, atrophy, IM, and dysplasia were distinguished. The subjects with normal histology or just only mild inflammation were classified as the normal group (NOR). Those with moderate-severe inflammation were divided into the gastric inflammation group (GI). Gastric atrophy, IM, and dysplasia usually occur in one section, and subjects with severe dysplasia more easily suffer from GC than those with mild and moderate dysplasia. To further explore the differences among gastric pathologies, we determined that the subjects with only gastric atrophy were classified as the gastric atrophy group (GA); those with either IM or mild-moderate dysplasia were classified together as the gastric premalignant lesion group (GPL); and those with severe dysplasia were divided into the gastric severe dysplasia group (GSD). Patients with GC were classified as the gastric cancer group (GC).

### 2.4. *IL-1B*-511 (rs 16944) Genotyping

DNA was extracted from gastric mucosal biopsies with Tissue/cell Genome DNA Extraction Kits (Yuanpinghao Biotech, Tianjin, China; 110901). PCR amplification was performed by PCR MIX Kits (Dongsheng Biotech, Guangdong, China; 068). Primer sequences were as follows: forward primer: 5′-GTTGTGTGAGCTTATCTCCAGG-3′; reverse primer: 5′-GGAATCTTCCCACTTACAGATGG-3′. PCR conditions were 94°C for 4 minutes, then 32 cycles of 94°C for 30 seconds, 60°C for 30 seconds, 72°C for 30 seconds, and finally 72°C for 10 minutes and 10°C for 30 seconds. The PCR products were separated by electrophoresis on 1% agarose gel with Goldview fluorescent dyes. The PCR amplification was successful if only one single band of 241 bp was shown. Direct sequencing of PCR products was carried out by the dideoxy-chain termination method.

### 2.5. Quantitative Real-Time Polymerase Chain Reaction (Q-PCR) Assay

Total RNA was extracted from the biopsy specimens using RNAiso Plus Kits (TaKaRa, AKA1202). cDNA was synthesized using a ReverTra Ace qPCR RT Kit (TOYOBO, FSQ-101). PCR amplification was performed with a three-step method using an ROX Reference Dye and SYBR Premix Ex TaqTM (TaKaRa, AK6006). Initial denaturation for cDNA was 95°C for 30 seconds. The amplification condition was 40 cycles of 95°C for 5 seconds, 60°C for 30 seconds, and 72°C for 1 minute. The melt condition was 95°C for 15 seconds, 65°C for 1 minute, and 95°C for 15 seconds. The sequences of primers were the same as those of the *IL-1B*-511 genotyping primers as shown above. The level of IL-1*β* mRNA was normalized with *β*-actin as an internal control and compared with the data of one normal subject using the 2-ΔΔC(t) method.

### 2.6. Statistical Methods

Stata 11.0 software was used for statistical analysis. All allelic distributions of *IL-1B*-511 were tested for deviations from their corresponding Hardy–Weinberg equilibrium. Age (per 10 years), gender, *H. pylori*, *IL-1B*-511 genotypes, and TCM syndrome distribution among gastric histopathological groups were summarized by proportions, and were examined using the chi-square test or Fisher exact probability test. Multinomial logistic regression estimated the adjusted (multivariable) relative risk ratio (RRs), 95% CIs, and *P* values. Because data did not obey normal distribution, levels of IL-1*β* mRNA between two groups were assessed by the Wilcoxon test. All values were two-sided, and statistical significance was defined as *P* < 0.05.

## 3. Results

### 3.1. Characteristics of Subjects

**Figure 1:**
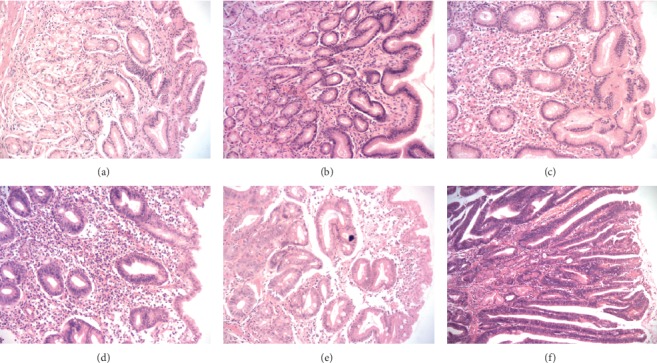
Representative images of different gastric histopathology (magnification, ×200): (a) normal gastric mucosa; (b) gastric mucosa with moderate inflammation; (c) atrophic gastric mucosa with mild-to-moderate inflammation; (d) atrophic gastric mucosa with severe inflammation and moderate dysplasia; (e) severe dysplasia with mild inflammation, moderate atrophy, and IM; and (f) gastric cancerous mucosa.

**Figure 2:**
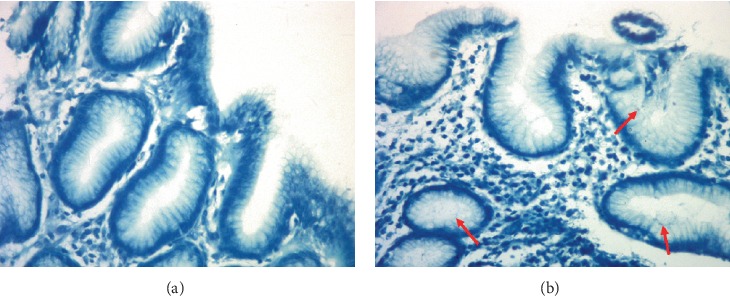
Representative images of *H. pylori* detection by methylene blue staining (magnification, ×400). (a) *H. pylori*-negative tissue and (b) *H. pylori*-positive tissue (*H. pylori* are present on the surface of gastric epithelial cells (red arrow)).

**Figure 3:**
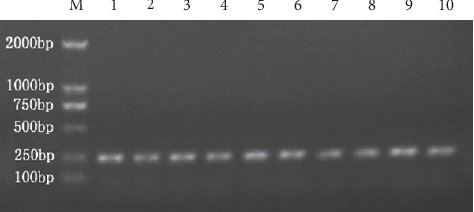
Agarose gel (1%) showing PCR-amplified fragments obtained by using primers developed by Taihe Biotechnology Company. Lane M is the marker; Lanes 1 to 10 are the DNA regions (241 bp) containing the *IL-1B*-511 (rs 16944) from 10 different individuals.

**Figure 4:**
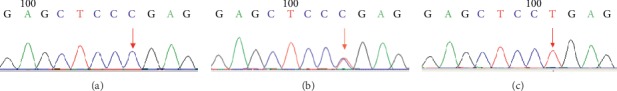
*IL-1B*-511 genotypes by the PCR-direct sequencing method. (a) CC genotype; (b) CT genotype; and (c) TT genotype.

The study subjects consisted of 464 participants (264 males and 200 females). The mean age was 46.26 ± 12.95 years. All of them were divided into six histopathology groups through assessment of gastric histopathology (Figure 1) and five TCM syndrome groups. *H. pylori* infection was examined by the rapid urease test and methylene blue staining (Figure 2). 78.66% (365/464) subjects were positive for *H. pylori* infection. *IL-1B*-511 genotypes in gastric mucosa from 424 subjects were detected by the PCR-direct sequencing method (Figures 3 and 4). The frequencies of genotypes CC, CT, and TT of *IL-1B*-511 were 22.41% (95/424), 53.54% (227/424), and 24.06% (102/424), respectively. The alleles at the individual locus did not deviate from the Hardy–Weinberg equilibrium in this study, with nonsignificant *χ*^*2*^ values (*χ*^*2*^ = 2.14, *P*=0.1435). In addition, levels of IL-1*β* mRNA in gastric mucosa were tested in 147 of 464 subjects (25 *H. pylori*-negative and 122 *H. pylori*-positive subjects).

### 3.2. Relationships between Gastric Histopathology and TCM Syndrome

**Figure 5:**
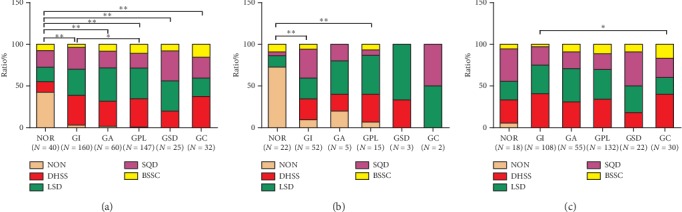
Relationship between gastric histopathology and TCM syndrome: (a) all subjects; (b) *H. pylori*-negative subjects; and (c) *H. pylori*-positive subjects. The vertical axis represents ratio of TCM syndrome distribution. Statistical significance was assessed by the chi-square test. The asterisk (^*∗*^) represents that *P* value is less than 0.05 and (^*∗*^^*∗*^) represents that *P* value is less than or equal to 0.001.

In all subjects, there were remarkable differences in frequencies of TCM syndrome between NOR group and other five histopathological groups (all *P* values were less than or equal to 0.001) and between the GI group and GPL group (*P*=0.025). Further analyses showed that subjects with nonsyndrome in NOR group were the most among gastric histopathological groups, and the proportion of BSSC syndrome in the GPL group was higher than that in the GI group (10.88% vs. 3.75%) (Figure 5(a)). To exclude the effects of *H. pylori* on gastric histopathology and TCM syndrome, we analyzed the distribution of TCM syndrome in different histopathological groups in *H. pylori*-negative and -positive subjects, respectively. In *H. pylori*-negative subjects, distribution of TCM syndrome in the NOR group was significantly different from that in the GI group (*P* < 0.001) and GPL group (*P* < 0.001), as well as that in the NOR group with *H. pylori* infection (*P* < 0.001) (Figures 5(b) and 5(c)). Proportion of *H. pylori*-negative subjects with nonsyndrome in the NOR group was much higher than that in the other histopathological group with or without *H. pylori* infection. Moreover, in *H. pylori*-positive subjects, significant difference of TCM syndrome distribution was observed between GI group and GC group (*P*=0.025) (Figure 5(c)). Specifically speaking, the rate of BSSC syndrome in the GC group was higher than that in the GI group (16.67% vs. 2.78%).

### 3.3. *IL-1B*-511 Genotypes and Gastric Histopathology/TCM Syndrome

Distributions of age, gender, *H. pylori* infection, and *IL-1B*-511 genotypes in different histopathological groups and TCM syndrome groups are listed in Tables 1 and 2. Significant differences included age and *H. pylori* among histopathological groups and TCM syndrome groups by the chi-square test or Fisher exact probability test (all *P* values were less than 0.001). Meanwhile, gender was significantly associated with TCM syndrome (*P*=0.008). However, there was no significant difference of frequencies of *IL-1B*-511 genotypes among gastric histopathological groups and TCM syndrome groups.

Considering the effects of cofounding factors, we performed multinomial logistic regression.  Table 3 described the outcome of the multinomial logistic regression using the NOR group as the reference group. Compared with the NOR group, every 10 years of age significantly increased the risk of GI (RR = 1.47), GPL (RR = 1.84), GSD (RR = 1.96), and GC (RR = 3.44). *H. pylori* infection dramatically elevated the risk of GI (RR = 2.89), GA (RR = 15.92), GPL (RR = 12.65), GSD (RR = 8.16,), and GC (RR = 18.39). Moreover, male gender significantly raised the risk of GI (RR = 2.19), GSD (RR = 3.35) and GC (RR = 4.35) after adjustment of age and *H. pylori*. However, when controlling age, gender, and *H. pylori* infection, there was still no association between *IL-1B*-511 genotypes and gastric histopathology.

To further evaluate roles of these variables on different histopathological stages, the latter histopathological group was compared with the former group, such as GI vs. NOR, GA vs. GI, GPL vs. GA, GSD vs. GPL, and GC vs. GSD by multinomial logistic regression. The results were shown in Table 4. Obviously, age, gender, and *H. pylori* had significantly important effects on gastric mucosa from normal to inflammatory (RRs were 1.47, 2.19, and 2.89). *H. pylori* infection was also a risk factor in gastric atrophy from inflammatory (RR = 5.51), while age played a crucial role in gastric severe dysplasia from gastric premalignant lesion (RR = 1.76). In the progress of gastric mucosa from atrophy to premalignant lesion, age might be a risk factor (RR = 1.29, *P*=0.052).

We used the NON group as a control to analyze relationships between covariables and TCM syndrome. As shown in Table 5, compared with the NON group, every 10 years of age significantly increased the risk of DHSS (RR = 2.80), LSD (RR = 2.80), SQD (RR = 3.06), and BSSC (RR = 4.32). *H. pylori* infection dramatically elevated the risk of DHSS (RR = 155.02), LSD (RR = 102.27), SQD (RR = 98.25), and BSSC (RR = 115.63). In addition, male gender was associated with an increased risk of DHSS (RR = 5.35), LSD (RR = 4.75) and BSSC (RR = 9.88). After adjusting with age, gender, and *H. pylori*, no significant relation was still found between *IL-1B*-511 genotypes and TCM syndrome in our study.

### 3.4. Relationships between Levels of IL-1*β* mRNA and Gastric Histopathology/TCM Syndrome

**Figure 6:**
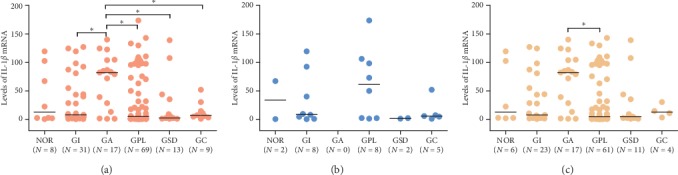
Effect of IL-1*β* expression on gastric mucosal pathology in (a) all subjects, (b) subjects without *H. pylori* infection, and (c) subjects with *H. pylori* infection. The lines represent medians. Statistical significance was assessed by the Wilcoxon test according to nonnormal distribution. The asterisk (^*∗*^) represents that the *P* value is less than 0.05.

In all subjects' detected levels of IL-1*β* mRNA in gastric mucosa, we demonstrated that IL-1*β* mRNA level in the GA group was dramatically higher than that in GI, GPL, GSD, and GC groups (All *P* values were less than 0.05) (Figure 6(a)). Also, in *H. pylori*-positive subjects, the median level of IL-1*β* mRNA in the GA group was the highest among histopathological groups and was significantly higher than that in the GPL group (82.14 vs. 4.63) (Figure 6(c)). However, no association between levels of IL-1*β* mRNA and gastric histopathology was observed in *H. pylori*-negative subjects (Figure 6(b)). These results suggested that *H. pylori*-induced gastric atrophy was associated with IL-1*β* overexpression.

**Figure 7:**
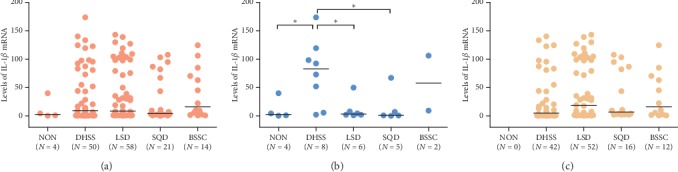
Effect of IL-1*β* expression on TCM syndrome in (a) all subjects, (b) subjects without *H. pylori* infection, and (c) subjects with *H. pylori* infection. The lines represent medians. Statistical significance was assessed by the Wilcoxon test. The asterisk (^*∗*^) represents that the *P* value is less than 0.05.

The relationship between IL-1*β* mRNA levels and TCM syndrome was also analyzed in our study. We found that in *H. pylori*-negative subjects, the median level of IL-1*β* mRNA in the DHSS group was significantly higher than that in the NON group (82.71 vs. 2.80; *P*=0.0272), LSD group (82.71 vs. 3.37; *P*=0.0142), and SQD group (82.71 vs. 1.06; *P*=0.0281) (Figure 7(b)). However, no significant differences of IL-1*β* mRNA levels were observed among different TCM syndrome groups in *H. pylori*-positive subjects (Figure 7(c)) and all subjects (Figure 7(a)). These results indicated that *H. pylori*-negative subjects with DHSS had a higher IL-1*β* expression.

## 4. Discussion

TCM, one of the oldest healing systems, plays an unreplaceable role in China and even the world. However, as the core of TCM, syndrome has been challenged by western medicine for a long time. It is essential to understand TCM syndrome from modern medicine and then to improve TCM. Nowadays, histopathology is the gold standard to diagnose various diseases and distinguish benign and malignant lesions. Both TCM syndrome and histopathology are the results of multiple factors acting on the host, reflect the current state of the host, and predict development of diseases. Thus, there may be a relationship between TCM syndrome and histopathology.

TCM theory thinks that patients with long-term disease will easily form blood stasis. In this study, we found that in *H. pylori*-positive subjects, BSSC syndrome was significantly more common in the GC group than that in the GI group, which suggests that endoscopy should be performed carefully for BSSC patients with *H. pylori* infection to observe their gastric mucosa, and medicine with activating blood should be used appropriately for GC patients with *H. pylori* infection. Interestingly, only 5.56% of *H. pylori*-positive subjects with normal gastric mucosa were differentiated to nonsyndrome, and 72.73% of *H. pylori*-negative subjects with normal gastric mucosa had nonsyndrome. The results indicate that syndrome possibly emerges before gastric pathology when one suffers from *H. pylori* infection, which provides an evidence for prevention before disease onset. In addition, not all *H. pylori* infection causes changes of gastric mucosa, which may be related to toxicity of *H. pylori* [21] and the host's state.

Both gastric pathology and TCM syndrome are results of multiple factors including host and environmental factors. In the present study, we focused on age, gender, *H. pylori*, *IL-1B*-511 genotypes, and IL-1*β* mRNA level which may participate in the formation of gastric pathology and TCM syndrome in subjects with *H. pylori*-related gastric diseases.

We showed that gastric histopathology and TCM syndrome were associated with age, gender, and *H. pylori*, but not *IL-1B*-511 genotypes. In detail, the risk of GI, GPL, GSD, and GC was increased with aging. Especially, age played an important role in the progress of gastric mucosa from normal to inflammatory and from GSD to GC. Although there was no statistical association, age had an increasing trend of gastric mucosa from GA to GPL. Moreover, *H. pylori* played a key role in the process of gastric mucosa from normal to inflammatory and from inflammatory to atrophy. These results are the same as those of Correa's model. For TCM syndrome, compared with the NON group, age and *H. pylori* infection increased the risk of DHSS, LSD, SQD, and BSSC, which indicates that the state of human body will be changed from balance to imbalance with age increasing and *H. pylori* infection. When controlling age and *H. pylori*, we found that male gender raised the risk of GI, GSD, and GC and played an important role in the gastric inflammation from normal gastric mucosa. Also, male gender elevated the risk of DHSS, LSD, and BSSC. These indicate gender may also be a factor in the formation of gastric pathology and TCM syndrome, which was probably related to sex hormones [22, 23] and their receptors [24, 25], as well as different lifestyle between males and females, such as smoking, frequent drinking, and bad dietary habits [26, 27]. For *IL-1B*-511 polymorphisms, TT genotype with *H. pylori* infection has been reported to increase the risk of gastric atrophy in high prevalence region of gastric cancer (Shanxi) in China [5] and the risk of GC in population with Han nationality (Hubei) [28], and TT/CT genotypes could increase risk of GC in Chinese population (Jiangsu [29] and Guangdong [30]). However, no significant association between *IL-1B*-511 genotypes and gastric histopathology/TCM syndrome was found whether adjustment of age, gender, and *H. pylori* or not in our study where most of the subjects were from Guangdong. Thus, more evidence and clinical factors need to be considered, such as haplotypes containing *IL-1B*-511 locus [7], lifestyle, and *H. pylori* strains [31].

We previously hypothesized that it was feasible to explore the formation mechanism of HPGD pathology and DHSS and other TCM syndromes from the inflammatory responses pathway and gastric microecology [32, 33] and found that interleukin-12 (IL-12), interferon-*γ* (IFN-*γ*), granulocyte-macrophage colony-stimulating factor (GM-CSF), and regulation upon activation normal T-cell expressed and secreted (RANTES) in gastric mucosa were involved in HPGD with DHSS pathology [34, 35]. In the present study, we examined levels of IL-1*β* mRNA in gastric mucosa. The results showed that IL-1*β* mRNA was overexpressed in *H. pylori*-induced gastric atrophy. IL-1*β* has been reported to induce parietal cells atrophy by suppressing Sonic Hedgehog (Shh) gene expression and the release of intracellular calcium [36]. Thus, we speculate that *H. pylori* may induce gastric atrophy by IL-1*β* overexpression. It has been reported that IL-1*β* protein level in serum of rats with DHSS syndrome was higher than that in serum of normal rats, and heat-clearing and dampness-eliminating prescriptions reduced expression of IL-1*β* and improved the symptoms and signs [37, 38]. This study also demonstrated that level of IL-1*β* mRNA in gastric mucosa of *H. pylori*-negative subjects with DHSS was significantly higher than those with nonsyndrome, LSD, and SQD. DHSS is thought be the status of struggle between the healthy qi and pathogenic qi, while nonsyndrome indicates the body is in a relative balance state, LSD is defined as dysfunction of the liver and stomach, and SQD is a pathological change characterized with impaired transporting and transforming function of the spleen. Thus, IL-1*β* overexpression might be a marker or a product of the host's struggle against pathogenic qi. Combined with our previous results, DHSS may be an external reflection of gastric mucosa inflammation.

## 5. Conclusions

In summary, we have shown that TCM syndrome occurs sooner than gastric pathological change after *H. pylori* infection. Under this condition, it is possible that TCM treatment helps prevent the development of gastric diseases. Our study also serves up an interesting perspective that combination of syndrome differentiation and *H. pylori* detection is likely helpful for predicting gastric histopathology to some extent. It remains unclear about the association of *IL-1B*-511 polymorphisms with gastric histopathology and TCM syndrome in patients with HPGD. But, we tentatively draw the conclusion that *H. pylori* may lead to gastric atrophy via enhancing IL-1*β* mRNA expression, and IL-1*β* mRNA overexpression in gastric mucosa may be one of the generality characteristics for *H. pylori*-negative subjects with DHSS.

## Figures and Tables

**Table 1:** Distribution of age, gender, *H. pylori*, and *IL-1B*-511 genotypes in gastric histopathological groups.

	All (*N* = 424) wt.%	NOR (*n* = 37) wt.%	GI (*n* = 147) wt.%	GA (*n* = 29) wt.%	GPL (*n* = 128) wt.%	GSD (*n* = 21) wt.%	GC (*n* = 32) wt.%	*P*
Age								**0.000**
<35 years	20.99	40.54	21.77	25.42	17.97	4.76	9.38	
35∼years	24.53	16.22	31.29	23.73	21.09	33.33	12.50	
45∼years	25.94	35.14	23.81	28.81	27.34	23.81	15.63	
55∼years	20.52	8.11	19.73	16.95	22.66	38.10	25.00	
65∼years	8.02	0.00	3.40	5.08	10.94	0.00	37.50	

Gender								0.118
Female	43.40	59.46	40.82	47.46	45.31	33.33	28.13	
Male	56.60	40.54	59.18	52.54	54.69	66.67	71.88	

*H. pylori*								**0.000**
Negative	22.17	59.46	33.33	8.47	10.16	14.29	6.25	
Positive	77.83	40.54	66.67	91.53	89.84	85.71	93.75	

*IL-1B*-511								0.875
CC	22.41	24.32	19.73	28.81	21.88	28.57	18.75	
CT	53.54	54.05	53.74	47.46	53.91	61.90	56.25	
TT	24.06	21.62	26.53	23.73	24.22	9.52	25.00	

**Table 2:** Distribution of age, gender, *H. pylori*, and *IL-1B*-511 genotypes in TCM syndrome groups.

	All (*N* = 424) wt.%	NON (*n* = 23) wt.%	DHSS (*n* = 134) wt.%	LSD (*n* = 140) wt.%	SQD (*n* = 97) wt.%	BSSC (*n* = 30) wt.%	*P*
Age							**0.000**
<35 years	20.99	60.87	19.40	20.71	17.53	10.00	
35∼years	24.53	8.70	29.85	25.00	22.68	16.67	
45∼years	25.94	30.43	20.90	27.14	28.87	30.00	
55∼years	20.52	0.00	23.88	20.00	21.65	20.00	
65∼years	8.02	0.00	5.97	7.14	9.28	23.33	

Gender							**0.008**
Female	43.40	65.22	38.06	41.43	53.61	26.67	
Male	56.60	34.78	61.94	58.57	46.39	73.33	

*H. pylori*							**0.000**
Negative	22.17	95.65	14.18	20.00	20.62	16.67	
Positive	77.83	4.35	85.82	80.00	79.38	83.33	

*IL-1B*-511							0.790
CC	22.41	17.39	22.39	23.57	20.62	26.67	
CT	53.54	47.83	50.75	54.29	59.79	46.67	
TT	24.06	34.78	26.87	22.14	19.59	26.67	

**Table 3:** Multinomial logistic regression of gastric histopathology compared with the NOR group.

	GI vs. NOR		GA vs. NOR		GPL vs. NOR		GSD vs. NOR		GC vs. NOR	
	RR (95% CI)	*P*	RR (95% CI)	*P*	RR (95% CI)	*P*	RR (95% CI)	*P*	RR (95% CI)	*P*
Age per 10 years	1.47 (1.03–2.06)	**0.033**	1.42 (0.96–2.12)	0.083	1.84 (1.28–2.66)	**0.001**	1.96 (1.20–3.20)	**0.007**	3.44 (2.12–5.57)	**0.000**

Gender										
Female	Ref.	—	Ref.	—	Ref.		Ref.		Ref.	—
Male	2.19 (1.01–4.73)	**0.046**	1.60 (0.66–3.92)	0.299	1.83 (0.81–4.13)	0.144	3.35 (1.04–10.79)	**0.043**	4.35 (1.45–13.06)	**0.009**

*H. pylori*										
Negative	Ref.	—	Ref.	—	Ref.	—	Ref.		Ref.	
positive	2.89 (1.35–6.16)	**0.006**	15.92 (5.11–49.61)	**0.000**	12.65 (5.18–30.87)	**0.000**	8.16 (1.99–33.38)	**0.004**	18.39 (3.64–92.90)	**0.000**

*IL-1B*-511										
CC	Ref.	—	Ref.	—	Ref.		Ref.		Ref.	—
CT	1.06 (0.42–2.77)	0.870	0.66 (0.23–1.91)	0.440	1.05 (0.39–2.82)	0.926	0.92 (0.25–3.44)	0.904	1.61 (0.42–6.09)	0.486
TT	1.61 (0.52–4.97)	0.409	1.07 (0.29–3.86)	0.922	1.51 (0.46–4.98)	0.500	0.43 (0.06–2.95)	0.387	1.96 (0.41–9.44)	0.401

**Table 4:** Multinomial logistic regression in cascade stages of HPGD.

	GI vs. NOR		GA vs. GI		GPL vs. GA		GSD vs. GPL		GC vs. GSD	
	RR (95% CI)	*P*	RR (95% CI)	*P*	RR (95% CI)	*P*	RR (95% CI)	*P*	RR (95% CI)	*P*
Age per 10 years	1.47 (1.03–2.06)	**0.033**	0.97 (0.75–1.26)	0.827	1.29 (1.00–1.68)	0.052	1.06 (0.72–1.57)	0.753	1.76 (1.08–2.86)	**0.024**

Gender										
Female	Ref.	—	Ref.	—	Ref.	—	Ref.		Ref.	
Male	2.19 (1.01–4.73)	**0.046**	0.73 (0.39–1.37)	0.333	1.14 (0.61–2.14)	0.609	1.83 (0.69–4.88)	0.228	1.30 (0.39–4.36)	0.673

*H. pylori*										
Negative	Ref.	—	Ref.	—	Ref.	—	Ref.		Ref.	
positive	2.89 (1.35–6.16)	**0.006**	5.51 (2.06–14.72)	**0.001**	0.79 (0.27–2.35)	0.678	0.65 (0.17–2.51)	0.527	2.25 (0.34–15.03)	0.401

*IL-1B*-511										
CC	Ref.	—	Ref.	—	Ref.	—	Ref.		Ref.	
CT	1.06 (0.42–2.77)	0.870	0.61 (0.28–1.29)	0.196	1.60 (0.75–3.39)	0.224	0.88 (0.30–2.57)	0.815	1.74 (0.44–6.83)	0.425
TT	1.61 (0.52–4.97)	0.409	0.66 (0.27–1.60)	0.362	1.42 (0.58–3.43)	0.442	0.28 (0.05–1.53)	0.142	4.61 (0.66–32.19)	0.124

**Table 5:** Multinomial logistic regression among different TCM syndromes.

	DHSS vs. NON		LSD vs. NON		SQD vs. NON		BSSC vs. NON	
	RR (95% CI)	*P*	RR (95% CI)	*P*	RR (95% CI)	*P*	RR (95% CI)	*P*
Age per 10 years	2.80 (1.53–5.12)	**0.001**	2.80 (1.54–5.10)	**0.001**	3.06 (1.67–5.60)	**0.000**	4.32 (2.22–8.40)	**0.000**

Gender								
Female	Ref.	—	Ref.	—	Ref.		Ref.	
Male	5.35 (1.54–18.59)	**0.008**	4.75 (1.38–16.32)	**0.013**	2.95 (0.84–10.32)	0.091	9.88 (2.30–42.37)	**0.002**

*H. pylori*								
Negative	Ref.	—	Ref.	—	Ref.		Ref.	
positive	155.02 (18.68–1286.50)	**0.000**	102.27 (12.53–834.84)	**0.000**	98.25 (11.85–814.74)	**0.000**	115.63 (11.67–1146.10)	**0.000**

*IL-1B*-511								
CC	Ref.	—	Ref.	—	Ref.		Ref.	
CT	0.46 (0.10–2.16)	0.325	0.47 (0.10–2.17)	0.334	0.62 (0.13–2.95)	0.549	0.39 (0.07–2.20)	0.284
TT	0.64 (0.12–3.31)	0.592	0.51 (0.10–2.58)	0.412	0.56 (0.11–3.00)	0.501	0.54 (0.08–3.56)	0.525

## Data Availability

The data used to support the findings of this study are available from the corresponding author upon request.
